# Identification of milk from different animal and plant sources by desorption electrospray ionisation high-resolution mass spectrometry (DESI-MS)

**DOI:** 10.1038/s41538-022-00129-3

**Published:** 2022-02-11

**Authors:** Yunhe Hong, Nicholas Birse, Brian Quinn, Holly Montgomery, Di Wu, Gonçalo Rosas da Silva, Saskia M. van Ruth, Christopher T. Elliott

**Affiliations:** 1 ASSET Technology Centre, Institute for Global Food Security, School of Biological Sciences, Queen’s University Belfast, Northern Ireland, UK; 2 Food Quality and Design Group, Wageningen University and Research, western, the Netherlands

**Keywords:** Mass spectrometry, Small molecules

## Abstract

This study used desorption electrospray ionisation mass spectrometry (DESI-MS) to analyse and detect and classify biomarkers in five different animal and plant sources of milk for the first time. A range of differences in terms of features was observed in the spectra of cow milk, goat milk, camel milk, soya milk, and oat milk. Chemometric modelling was then used to classify the mass spectra data, enabling unique or significant markers for each milk source to be identified. The classification of different milk sources was achieved with a cross-validation percentage rate of 100% through linear discriminate analysis (LDA) with high sensitivity to adulteration (0.1–5% v/v). The DESI-MS results from the milk samples analysed show the methodology to have high classification accuracy, and in the absence of complex sample clean-up which is often associated with authenticity testing, to be a rapid and efficient approach for milk fraud control.

## Introduction

Milk and dairy products are nutrient-dense foods that are relatively low in calorific content and provide high amounts of essential nutrients^1,2^. Many western cultures have been encouraged to eat or drink several servings a day, with the 2015 Dietary Guidelines for Americans recommending that adults consume 500–750 mL of milk or equivalent dairy foods per day^3^. However, avoidance of dairy products is found in some populations, a result of medical conditions, lifestyle choices, ethical considerations, or limited access to safe dairy products. Cow’s milk allergy (CMA) is an example of a medical condition that results in the avoidance of dairy products; CMA is the most common food allergy in young children, with approximately 2–3% of young children living in the developed world suffering from it^4^. Lactose intolerance is another medical condition for which people will attempt to avoid dairy products or find adequate replacements^5^. Evidence shows that dairy products from goats and sheep not only maintain the key nutritional features of cow milk, but are also easier to digest^6,7^. This has been cited to support the use of goat milk and sheep milk as more suitable alternatives to cow milk-based dairy produce^8^. Camel milk is also described as an alternative to cow milk as it lacks β-lactoglobulin, which is often responsible for cow milk allergy, and lower levels of casomorphin present are thought to assist in the metabolism of lactose, leading to lower intolerance in consumers^9,10^. Plant-based milks, such as oat milk and soya milk form a large part of the dairy-free products category. Vegetarians and vegan consumers may find that plant-based milk products are their only option. Lifestyle and ethical considerations have resulted in dairy-free milk alternatives rapidly increasing in popularity with consumers in recent years, resulting in an increasingly diversified consumer market. These alternative dairy products can no longer be considered niche products, indeed, in the UK, non-dairy milk products have been used to track inflation since 2017.

The UK milk market and wider grocery market has noticeably changed as a result of the COVID-19 pandemic^11^. The difficulties in managing yield variations in cow milk alternative products together with the higher prices resulting from both increasing demand and constrained supply risks the fraudulent use of cow milk in milk from different animal and plant sources. Milk adulteration has been a subject of concern for a number of years; adulterants can range from low-cost cow milk being used to bulk out high-price milk products to chemical additives such as melamine. These adulterants can have serious adverse health effects, yet due to the profitability, absence of adequate monitoring, and lack of proper law enforcement, adulteration fraud is thought to be highly prevalent within the sector^12,13^.

Inadvertent adulteration or contamination with potential allergens can also occur when several animal species’ milks are handled on the same manufacturing equipment^14^. CMA patients or lactose intolerant consumers may suffer severe adverse health effects after ingesting goat milk or plant-based milk which is adulterated with cow milk, but the reason behind the adulteration, whether deliberate or accidental is inconsequential to the health effects to the consumer^15^. Montgomery et al collected safety and fraud reports for milk and milk products from the online RASFF portal, finding there were a total of 355 notifications relating to milk and milk products over the last five years, they then provided a summary of fraud relating to these products over the same period. Their analyses indicate that although the number of fraud incidents was smaller, they still pose a very significant risk to human health^16^.

For milk to be sold, unfortunately, the processes behind milk adulteration have become sufficiently sophisticated and widespread that regulatory bodies may find adulteration detection difficult or impossible^16,17^. Focusing on milk adulteration, it is easy to find the maximum residue limits (MRLs) and tolerable daily intake (TDI) for known chemical contaminants, such as melamine^18^, as well as plasticizers, preservatives, and antimicrobials, all of which are of significant concern for their impacts on human health^19,20^ and to adulterate or dilute samples down in such a way as to defeat existing testing techniques. The weakest link in milk fraud identification is arguably the lack of methods for detecting and analysing adulteration from different species. There are relatively few government regulations and reference methods relating to milk species adulteration. The current European Community reference method for cow milk identification is based on isoelectric focusing (IEF) of β- and γ-caseins, since both β-casein and γ-casein from bovine milk contain immunoglobulin E (IgE)-binding epitopes^21^. This technique requires complex sample preparation and lengthy assay times, which are far from ideal in a commodity such as milk with a short shelf-life and rapid distribution from farm to retailer. Further studies have also shown that there are several limitations to the current EU official milk adulteration evaluating method, such as false-positive results when it is performed on water buffalo β-CN (f100-207)^22^.

A variety of different methods have been trialled as alternatives to the European reference method for milk species differentiation, with DNA-based techniques favoured for species identification. Applications of PCR were used to investigate the adulteration of goat milk produced by smallholders with bovine milk as an adulterant^23^. Several studies have confirmed the potential of DNA-based methods for detecting the fraudulent admixture of milk from different species in milk and milk products^24,25^. Nevertheless, each sample must undergo complex and time-consuming preparation procedures, such as DNA isolation and quantification, and the design of species-specific primers^26^. Moreover, PCR is an indirect method and can suffer from DNA contamination due to the amplification of minor components^27^.

Recent studies have presented detection methods based around high resolution mass spectrometry which can undertake speciation, a capability that is potentially valuable when attempting to detect the presence of cow milk in putatively other milk species^28–30^ or plant proteins in raw milk^31,32^. Wei Jia et al. reported that high-resolution mass spectrometry can provide an efficient approach for the discrimination of milk from different mammalian species by untargeted analysis of small molecules found within the sample, an approach known as ‘Foodomics’^33^. Innovation in new ionisation sources, and the ongoing development of existing technologies provide ever more possibilities for the identification of milk fraud. Matrix-assisted laser desorption/ionisation-time of flight mass spectrometry (MALDI–TOF MS) has been adapted to profile differences in milk chemical compounds from different mammalian species^34–36^ and for the discrimination of plant-based milk from cow milk. However, sample pre-treatment and MALDI matrix chromophore preparation procedures are complex, which is an issue when attempting to develop rapid MALDI screening workflows.

The focus on food security research in recent years has been to detect issues when they occur, whilst simultaneously improving processes to try and eliminate issues from occurring in the first place^37^. Nascimento et al. evaluated the assay methods used by regulatory agencies throughout the World, and upon observing the limited access to mass spectrometry that exists, predicted that the development of inexpensive alternatives to mass spectrometry would continue, resulting in ever faster and more environmentally friendly in situ tests^38^.

Ambient mass spectrometry is a small but growing area, and has been widely used in food research in recent years^39,40^. Ambient mass spectrometry is designed to remove much of the complexity inherent in existing mass spectrometry techniques, such as eliminating chromatography, reducing or eliminating the need for the ion source to operate under complex vacuum or temperature conditions, and enabling direct sampling in close proximity to the instrument. This enables the use of smaller, less expensive, and potentially portable mass spectrometers, features which make the technique particularly well suited to in-situ or on-site testing in the agri-food sector^41^. DART, a well-known AMS ion source, is a strong tool for milk fraud analysis. Zhang et al.^42^ demonstrated a technique for fast detection of dicyandiamide (DCD) in powdered milk using DART/Q-TOF. Hrbek et al.^43^ devised a DART–HRMS approach for authenticating milk and milk-based goods, which permitted differentiating milk mixes manufactured at a 50% (v/v) adulteration level. However, pre-treatment procedures such as organic solvent extraction and centrifugation are utilised in many experiments, lowering the method’s throughput, and expensive consumables were still used, which can be avoided with DESI, making the total cost per sample lower with DESI.

There are a wide number of different ambient mass spectrometry techniques which have been developed since the initial techniques of desorption electrospray ionisation (DESI) were first commercialised in 2005^44,45^. DESI-MS, which is aimed at the analysis of sample surfaces and tissues, is undertaken at ambient atmospheric pressures^46,47^. The electrical charge is contained in an electrospray solvent mist (primary ionisation) which causes secondary ionisation to occur at atmospheric pressures^47^. DESI is a minimally destructive ionisation technique and typically known for the ionisation of small molecules in singly charged forms, although it has also been demonstrated in protein and amino acid analysis^39,46–49^. The utilisation of DESI-MS for the detection of protein and peptides directly from a tissue section, a process known as mass spectrometry imaging (MSI), can be seen as an avenue of investigation complimentary to MALDI and other MSI techniques^48,50^.

DESI is a soft ionisation technique, causing little or no fragmentation of the target analyte. This makes the technique a strong candidate for samples with labile analytes, such as milk, and is one key reason why it has found such widespread usage.

The advantages of using DESI-MS for rapid accurate classification of milk samples are: (a) no need for organic solvent use during sample treatment, with water-diluted milk samples being directly loaded onto the glass slide sample plate, keeping components in milk as unaltered as possible; (b) soft, non-destructive ionisation similarly contributes to the structural integrity of the analytes, and samples can thus be run repeatedly;^51^ (c) no need for sample clean-up, since satisfactory spectral data can be obtained from diluted samples; d) the analysis time is approximately 10 s. In addition, the considerably reduced consumption of organic solvent in analyses demonstrated that DESI-MS is a much more environmentally friendly assay method, whilst the use of DESI-MS in combination with a high-resolution time of flight instrument can potentially allow a virtually instant change from using the system for rapid screening to using the system for detailed, in-depth analysis of samples of concern.

The objective of this study was to develop a reliable, sensitive, and rapid assay method capable of the identification of milk from different animal and plant sources. A DESI source was coupled with a quadrupole-time of flight (Q-ToF) system for milk classification and biomarker identification. The classification/prediction models based on principal component analysis (PCA)/LDA were built to identify milk species adulteration. This research focused on using DESI-MS to identify stable lipids used as biomarkers for cow milk in types of non-cow-milk claiming, with a detection limit of cow milk content of 0.1–5%. The adulteration of different milk species can be identified by using non-destructive testing whilst keeping the milk samples as close to retail conditions as possible during the whole assay procedure. The proposed method is simple, accurate, time-saving, and environmentally-friendly, providing a reliable and fast method for investigating the prevalence of mislabelling.

## Results and discussion

### Method development

The sample treatment procedure was optimised in order to obtain a suitable MS intensity. Three different scenarios were considered at this stage: (1) direct analysis, (2) milk diluted with methanol, (3) milk diluted with deionised water. The natural thickness and density of milk could potentially cause several problems by direct analysis. A thick sample cannot remain on the glass slide sample plate due to surface tension. It is also difficult to evaporate aqueous matrixes at room temperature. Thus, direct analysis was not considered for this study. The use of methanol or acetonitrile for milk sample pre-treatment is a common method for mass analysis.

**Fig. 1:**
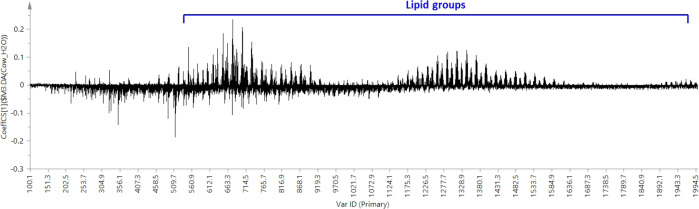
The comparison of sample treatment differences. Coefficients for different sample treatment procedures, milk treated with methanol = −1, milk treated with pure water = 1.

The main purpose of using organic solvents during sample treatment is to dilute or clean-up samples for targeted analysis, diluting potential contaminants which may interfere with the analytes of interest or the analysis itself. However, this can cause unsatisfactory results in untargeted analysis by diluting or removing compounds that may later prove to be useful as biomarkers or have some other utility in assessing the quality or safety of the sample. The solvent addition can also increase ionisation potentials for many compounds in the milk matrix, potentially increasing the fragmentation of compounds at the time of initial ionisation, and reducing the formation of water clusters, which will further change the ionisation behaviour of lipids. As can be seen from the binary comparison diagram in Fig. 1, the content of compounds with smaller molecular weight is more abundant when use methanol. This situation may also because milk precipitation caused by methanol is removed by centrifugation, caused big loss of features. The results of the chemical compound difference between using methanol and water as sample treatment solvent are shown in Fig. 1. From the mass spectra of different sample treatment procedures, most lipid groups in cow milk were removed by using an organic solvent (methanol), which means a large number of biomarkers are lost before instrument data acquire. Thus, the use of water as a solvent was considered as the best method for this study.

The procedure was optimised by the use of different dilution ratios of water/milk. The TIC intensity increases as the ratio of water rises. Milk: water = 1:4 (v: v) was found to be the dilution that gave the greatest sensitivity. This was consistent with the results reported in the prior literature^34^. A 2 µL aliquot of sample solution (0.4 µL milk:1.6 µL water) was directly loaded onto the glass slide sample plate and evaporated to dryness at room temperature for DESI-MS analysis.

The analysis time per sample was similarly assessed experimentally and an optimal time of 15 s acquisition time per sample was determined, giving a total time to analyse 96 samples of approximately 25 min. This compares very favourably with LC-MS where rapid methods of 5 min per sample would only enable five samples within the same timeframe. The sample preparation and drying time was comparable to preparing samples for a dilute and shoot LC-MS method and considerably faster than many more complicated sample preparation steps^52^.

To evaluate the”within-group” milk difference and validate the milk species models, the production process and farm location were considered as the main factors^53,54^. To maximise the validation coverage, cow milk samples were sourced from a total of 30 different farms and production systems, including a mixture of UHT and pasteurised milks (totalling 103 number of cow’s milk samples). Cow milk was used to indicate the stability and reliability of lipids as biomarkers of cow milk presence in alternative cow milk products. Goat milk was sourced from two different farms (five semi-skim milk samples and 22 whole milk samples). Thirty-six camel milk samples were sourced from two online distributors and had five different production dates. Oat milk samples were sourced from four different factory suppliers (totalling 34 number of samples); both of these are original oat milk, finally, soya milk samples were sourced from five different suppliers (comprising eight no sugars, six unsweetened, and 59 unsweetened).

**Fig. 2:**
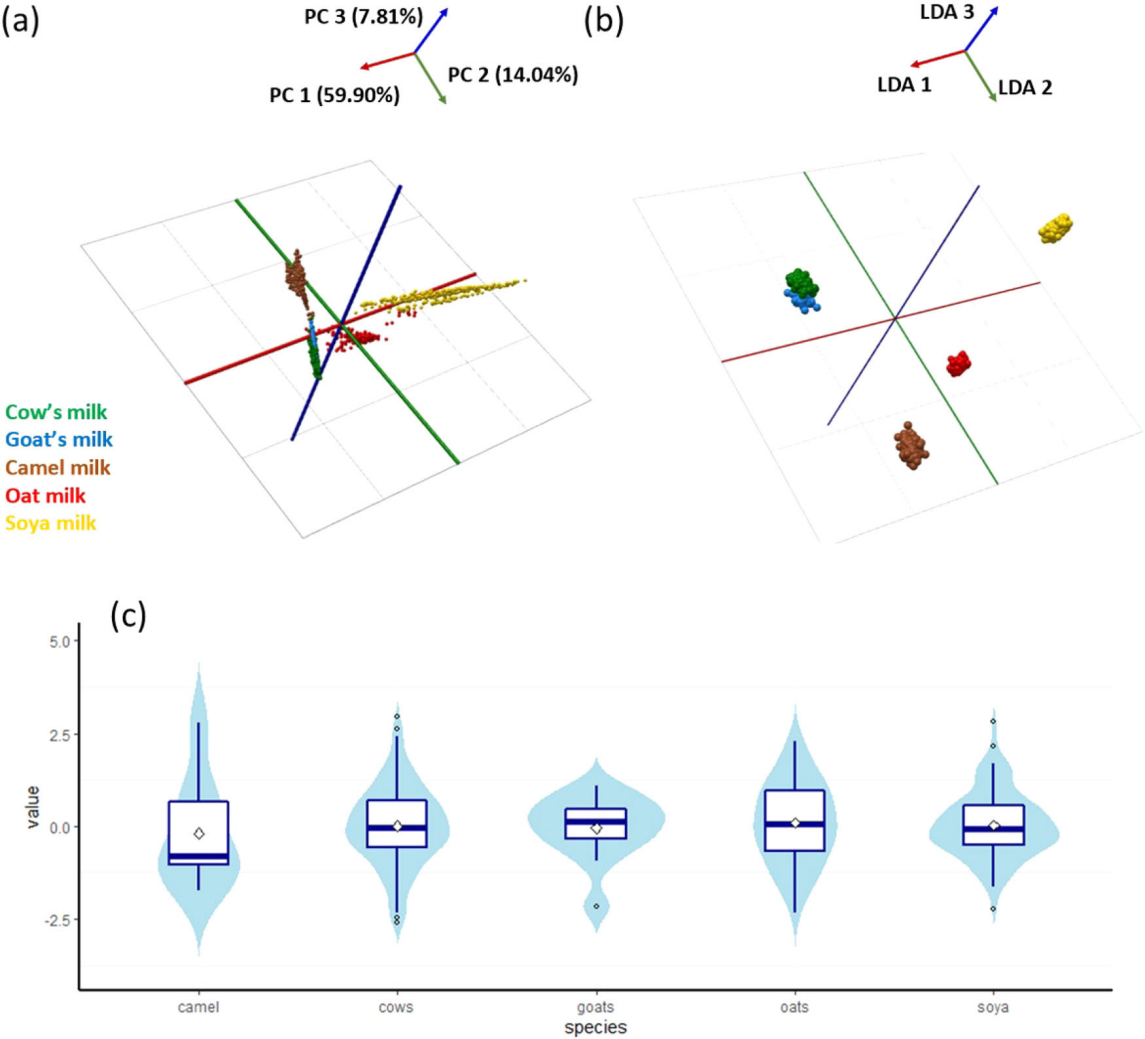
Main effects of within-group differences on milk species identification. **a** PCA score plot and **b** LDA plot of DESI-MS spectral data (m/z 100–20,00) obtained from five milk species (different production procedure and farm location inside each group). **c** Violin and box plots for the distribution of validation scores. There was no statistical significance among the measurements of all the five milk species (*p* < 0.01). Symbols indicate outliers, which show the discrete degree of data.

The unsupervised PCA model clearly shows separation between all five classes of milk products, albeit with only limited separation between cow milk and goat milk. Other types of milk, including the plant-based products show much clearer separation (Fig. 2a). The supervised LDA model shows clearer separation between all five classes, albeit still with cow milk and goat milk in close proximity to each other (Fig. 2b).

R (version 4.0.5; ggplot2 and tidyverse package) was used to plot data distribution. The distribution of values is shown in Fig. 2c. Each box-plot is surrounded by a violin plot representing data density, which is centred at the mean value. The accuracy is assessed for within-group differences of complex models in the validation set. Horizontal blue lines represent median values. The diamond box indicates the mean values for each group. Compared with different milk species, the difference within cow milk samples was minor. Yet, the differences between each milk species group were highly significant.

### Identification of cow milk in non-dairy milk

**Fig. 3:**
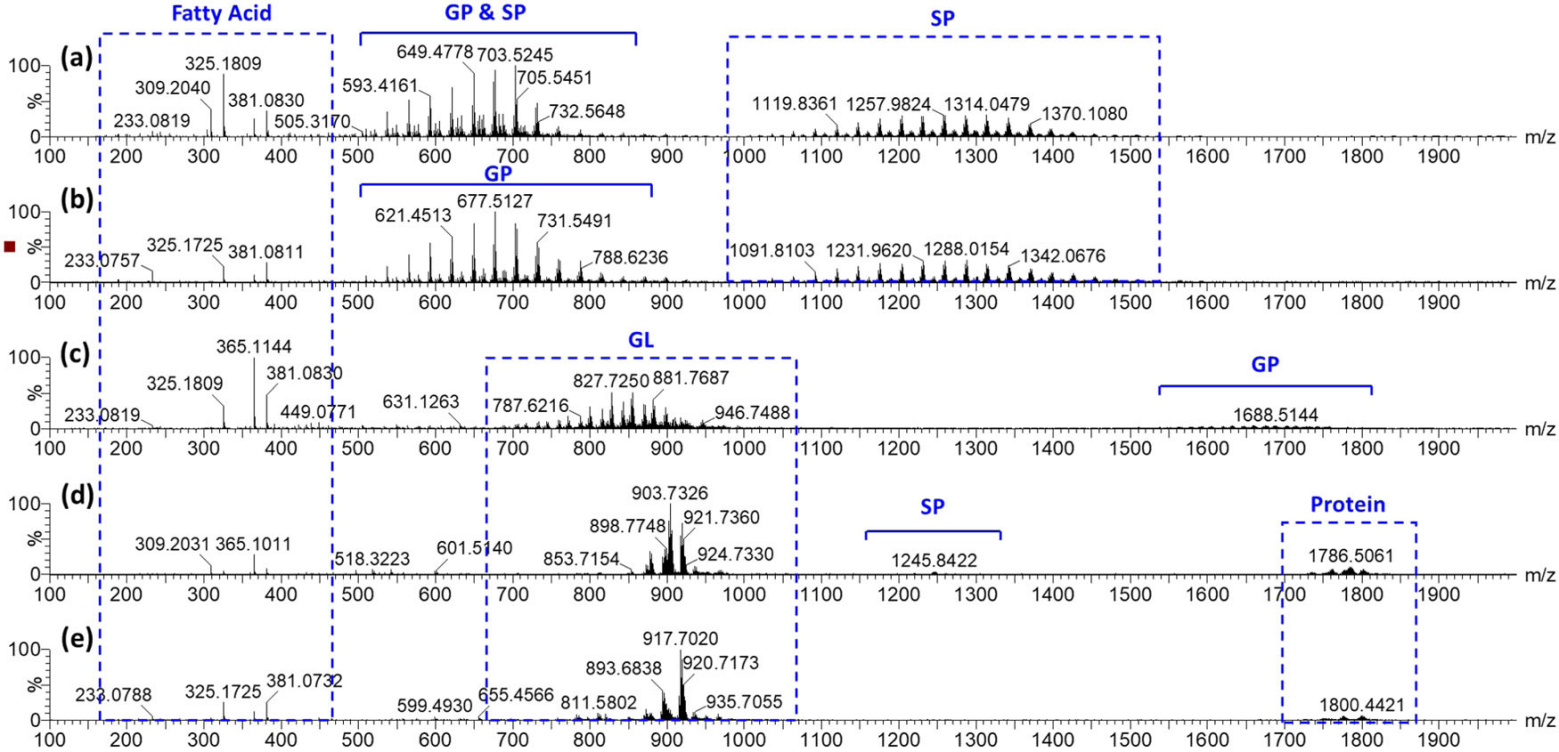
Mass spectra. **a** cow milk, **b** goat milk, **c** camel milk, **d** oat milk, and **e** soya milk samples with DESI-MS.

Figure 3 shows the mass spectra of five different species of milk samples by using DESI-MS. The analysis of cow milk and goat milk was found to yield spectra dominated by fatty acids, glycerophospholipids (GP), and sphingolipids (SP). There are significant differences in the spectral features for cow milk and goat milk, as expected when reviewing the PCA and LDA plots, but upon further investigation, characteristic differences can be observed as well. This is in contrast to cow milk, which shows significant differences in spectral features to those observed in camel milk, oat milk, and soya milk spectra. Glycerolipid (GL) groups were also found to differ between cow milk and camel milk, oat milk, and soya milk samples. GP group (GP1501) was found in camel milk, and SP group (SP0303) was found in oat milk. A small number of protein groups were also found in oat milk and soya milk, potentially serving as biomarkers.

**Fig. 4:**
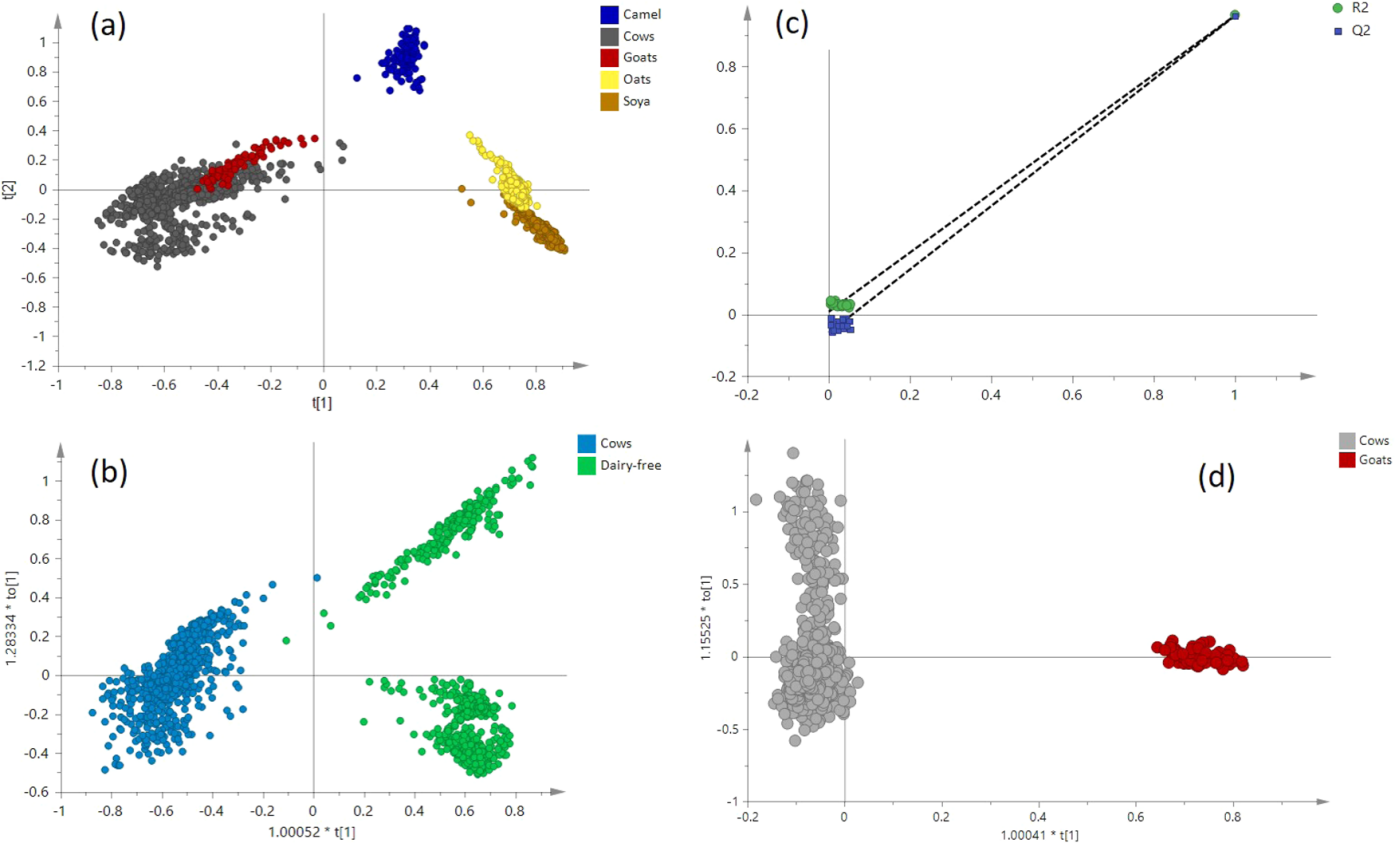
Differentially component analysis between different milk species. **a** PCA of five milk species; **b** OPLS-DA between cow’s milk and other milk species; **c** Permutations plot of OPLS-DA model; **d** OPLS-DA between cow’s milk and goat’s milk.

Unsupervised PCA and Orthogonal Partial Least Squares (OPLS-DA) algorithms for dimensionality reduction were used to analyse the features of each milk type (Fig. 4). The lipid groups of milk samples from the five different types were acquired in positive mode. The PCA score plot shows the chemical compounds of each milk type, suggesting that there are significant biochemical differences between them (Fig. 4a). R2X and Q2 values of 0.898 and 0.867 were obtained suggesting that the PCA model was both robust and had good predictive ability towards additional data points. The classification performance resulted in 100% separation between cow milk and other milk species.

An OPLS-DA model was employed to sharpen the established separation. As expected, superior separation of cow milk and other milk species was observed in the score plot of OPLS-DA (Fig. 4b). Goat milk was somewhat more difficult to differentiate from cow milk, but by using a binary model, it was also clearly recognisable (Fig. 4d). 103 cow milk, 36 camel milk, 27 goat milk, 34 oat milk, and 73 soya milk samples were obtained (6–9 parallel repeat data acquisition for each sample). A multivariate model was built from all sampling points, a total of 2225 spectra including 940 spectra from the cow milk group, 516 spectra from the camel milk group, 96 spectra from the goat milk group, 230 spectra from the oat milk group, and 443 spectra from the soya milk group. In order to evaluate the credibility of this OPLS-DA supervised analysis, a permutations plot was used to assess the integrity of OPLS-DA model. Validation of the accuracy and reliability of the model was performed with the parameters of R2Y and Q2. Q2 was used to evaluate the statistical quality of the model, and determine the fraction of Y variation that could be predicted, while R2Y was used to assess the degree of adjustment from the Y variance explained by the model. The more R2Y and Q2 values approach 1, the higher the model’s predictive power. When the R2Y and Q2 are above 0.5, the model is considered to have strong predictive properties^55^.

The plot in Fig. 4c shows that Q2-values to the left all lower than the original Y level. Meanwhile, R2-values also show promise. This indicates that the model has a high capability to explain the sample differences (R2Y = 0.965 and Q2Y = 0.964 in positive ion mode).

### Candidate biomarkers

**Fig. 5:**
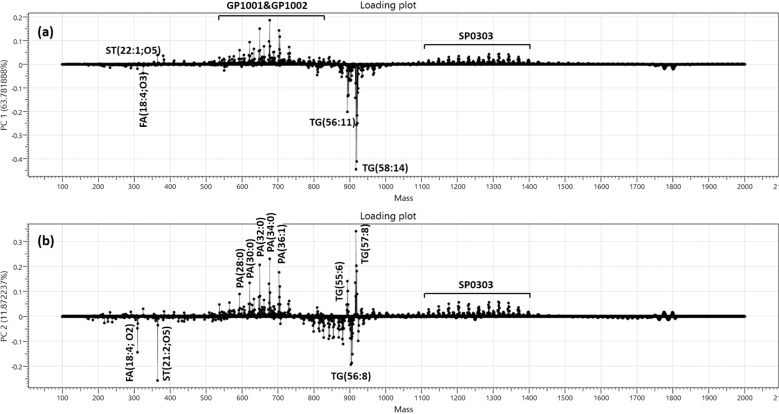
The principal component loading plot shows the separation between different milk species. **a** First principal component loading plot, **b** second principal component loading plot.

Under PCA, the individual principal component composition was elucidated by generating loading plots. For mass spectrometric data, the loading functions show what the contribution of individual mass spectrometric peaks is to the given principal component (PC) (Fig. 5). The loading function responsible for the separation of the lipidomic profiles of cow milk and other milk species shows clear differences between those two classes. The separation of the DESI lipidomic profiles of cow milk, goat milk, camel milk, soya milk, and oat milk, again shows clear differences between those classes.

The first principal component (PC1) contributed to 63.78% of the total explained variations (Fig. 5a), and the second principal component has 11.87% contribution in the total explained variations (Fig. 5b). Cow milk and goat milk contain more lipid types, like glycerophospholipids (GP1001 and GP1002 lipid group) and sphingolipids (SP0303). Whereas camel milk, oat milk, and soya milk tend to produce glycerolipids groups, for example, TG(55:6), TG(56:8), TG(56:11), TG(57:8), and TG(58:14). A small number of Fatty acids (FA(18:4;O2), FA(18:4;O3)) and sterol lipids (ST(21:2;O5), ST(22:1;O5)) are also abundant, as revealed by the loading plot and MS/MS fragmentation of the corresponding ions.

**Fig. 6:**
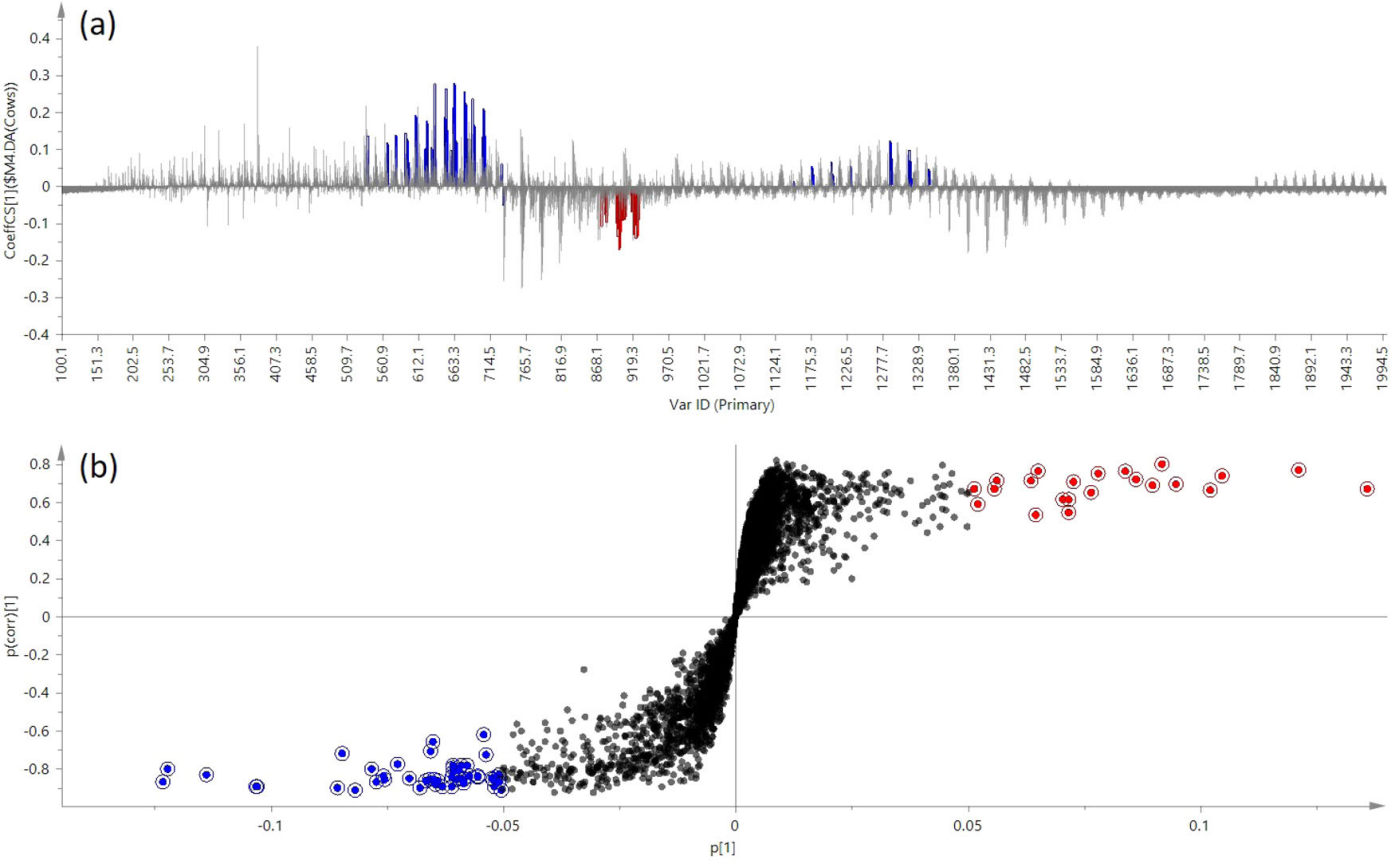
**a** Coefficients for different milk groups. Cow milk = -1, other milk species = 1; **b** Candidate biomarkers marked in OPLS-DA/S-plot of ions responsible for the cow milk classification found at the bottom of the plot, whereas ions responsible for dairy-free samples were located at the top.

Differential lipidomics made it possible to identify several candidate biomarkers for different milk groups. Results showed 9501 components in total belonging to these 5 milk species, with the mass bin set in 0.2 Da in order to make data sizes manageable while accurately separating metabolites. The differential components of cow milk and other milk species are displayed in Fig. 6. The corresponding S-plot values and t-tests were used to assay the statistical significance between different milk species (Fig. 6b), with the ions with high variable importance being responsible for discriminating cow milk and non-dairy milk (Fig. 6a). The red points are the candidate biomarkers from the cow milk alternative group (positive quadrant), blue points are the candidate biomarkers from the cow milk group (negative quadrant). Both sets were selected due to their high reliability [|*p*(corr)| > 0.5], and high influence on the model (|*p*| > 0.05). The lipid groups match clearly in the coefficient plot (Fig. 6b).

As shown in the S-plot, a total of 28 known robust candidate markers (Table 1) enabled the differentiation between different milk species (23 from cow milk, five from other milk species). The lipids were identified by MS scan data for the lipids and searched against lipid groups available from the LipidMaps database. Lipid marker candidates were then evaluated using MS/MS data for chemical structure confirmation. The lipid composition varies between different milk species, with glycerophospholipids (GP), and sphingolipids (SP) being more abundant in the cow milk components. Biomarkers of cow milk all belong to GP1001, GP1003, SP0203, and SP0303 lipid groups. Most of the assigned lipid identifications from other milk species are glycerolipid (GL) species, belonging to the triacylglycerols [GL0301] sub/class.

**Table 1:** List of ions identified based on the contribution of each ion to the variance of the observations and their reliability.

Milk type	Mass bin [m/z]	S-plot *p*[1] value	S-plot *p*(corr) value	Representative mass [m/z]	Accurate mass [m/z]	Formula	Lipid identifier	LM ID
Cow milk	537.3	−0.066	−0.854	537.3516	537.3551	C27H53O8P	PA(24:0)	LMGP10010030
	565.3	−0.070	−0.853	565.3904	565.3864	C29H57O8P	PA(26:0)	LMGP10010933
	577.5	−0.055	−0.836	577.4272	577.4228	C31H61O7P	PA(P-28:0)	LMGP10030001
	593.5	−0.086	−0.898	593.4140	593.4177	C31H61O8P	PA(28:0)	LMGP10010944
	605.5	−0.061	−0.892	605.4564	605.4541	C33H65O7P	PA(P-30:0)	LMGP10030003
	619.5	−0.068	−0.903	619.4680	619.4697	C34H67O7P	PA(P-31:0)	LMGP10030005
	621.5	−0.103	−0.895	621.4513	621.4490	C33H65O8P	PA(30:0)	LMGP10010943
	628.5	−0.065	−0.656	628.5848	628.5874	C38H77NO5	Cer(38:0;O4)	LMSP02030017
	633.5	−0.075	−0.854	633.4876	633.4854	C35H69O7P	PA(P-32:0)	LMGP10030007
	647.5	−0.082	−0.915	647.5047	647.5010	C36H71O7P	PA(P-33:0)	LMGP10030009
	656.5	−0.054	−0.621	656.5998	656.6187	C40H81NO5	Cer(40:0;O4)	LMSP02030018
	661.5	−0.073	−0.779	661.5192	661.5167	C37H73O7P	PA(P-34:0)	LMGP10030012
	677.5	−0.122	−0.803	677.5127	677.5116	C37H73O8P	PA(34:0)	LMGP10010940
	687.5	−0.061	−0.793	687.5358	687.5323	C39H75O7P	PA(P-36:1)	LMGP10030019
	703.5	−0.114	−0.835	703.5297	703.5272	C39H75O8P	PA(36:1)	LMGP10010214
	731.5	−0.066	−0.705	731.5517	731.5585	C41H79O8P	PA(38:1)	LMGP10010220
	1146.5	−0.057	−0.840	1146.5184	1146.5087	C47H86N7O17P3S	CoA(26:0)	LMFA07050327
	1174.5	−0.052	−0.844	1174.5574	1174.5400	C49H90N7O17P3S	CoA(28:0)	LMFA07050351
	1202.7	−0.059	−0.856	1202.6506	1202.6211	C52H101NO25P2	M(IP)2C(34:0;O3)	LMSP03030108
	1232.7	−0.061	−0.845	1230.6704	1230.6524	C54H105NO25P2	M(IP)2C(36:0;O3)	LMSP03030109
	1286.7	−0.058	−0.875	1286.7344	1286.7150	C58H113NO25P2	M(IP)2C(40:0;O3)	LMSP03030111
	1314.7	−0.065	−0.854	1314.7518	1314.7463	C60H117NO25P2	M(IP)2C(42:0;O3)	LMSP03030112
	1342.7	−0.059	−0.807	1342.7896	1342.7776	C62H121NO25P2	M(IP)2C(44:0;O3)	LMSP03030113
Oat milk	872.7	0.056	0.711	872.7094	872.7103	C50H98NO8P	PC(42:1)	LMGP01012161
	903.7	0.072	0.544	903.7435	903.7436	C59H98O6	TG(56:8)	LMGL03016229
Oat milk Soya milk	917.7	0.136	0.671	917.7568	917.7593	C60H100O6	TG(57:8)	LMGL03016407
Soya milk	893.7	0.095	0.692	893.7489	893.7593	C58H100O6	TG(55:6)	LMGL03015620
Camel milk	881.7	0.050	0.654	881.7578	881.7593	C57H100O6	TG(54:5)	LMGL03010352

Identities confirmed by MS/MS ion fragmentation analyses.

**Fig. 7:**
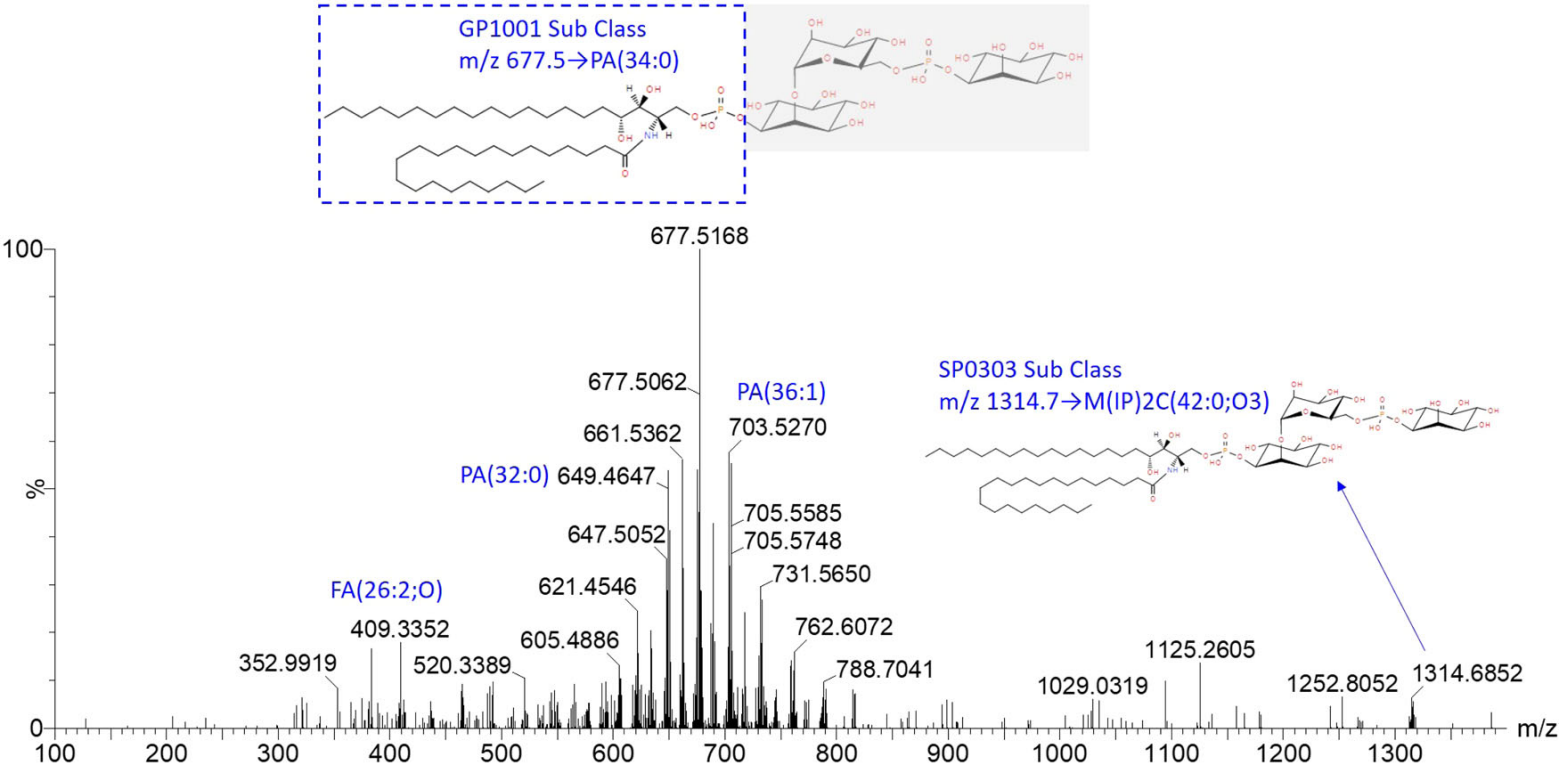
Fragmentation spectra of M(IP)2C(42:0;O3) (m/z 1314.7463).

The process for characterising biomarkers from different milk species is displayed in Fig. 7. for example, the peak at m/z 1314.7 when fragmented at a collision energy of 20 V produced daughter (fragment) ions at m/z 677.5, 703.5, 649.5 that correspond to PA(32:0), PA(34:0), PA(34:0), and further components from the GP1001 group, due to the obvious fracture site of the compound. This finding was confirmed with GP1001 compositions presenting at higher levels of mass intensity. Fatty acid compositions were found in most of these biomarker MS/MS mass spectra, due to the chemical structure of these FA compositions.

Thus, lipids specific to cow milk were identified through high-resolution mass analysis using a DESI-MS system approach. The possible molecular formula and structures of the markers were calculated by high-accuracy quasi-molecular ions. GP1001, GP1003, and SP0202 lipid groups can be set as dairy milk biomarkers for identifying mislabelled non-cow milk samples.

### Adulteration analysis of milk samples

**Fig. 8:**
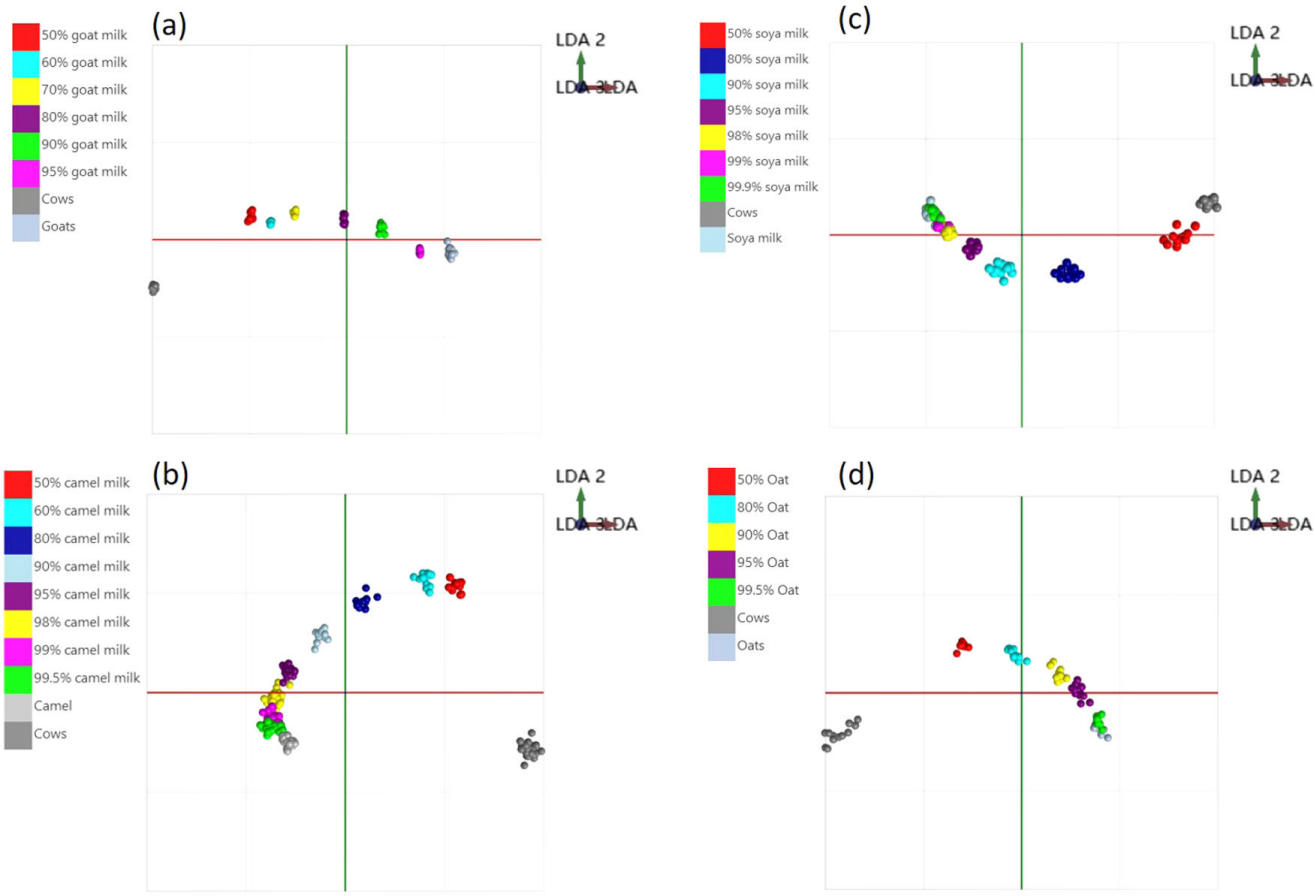
LDA analysis of different milk species adulterated with different milk species. Goat milk (**a**), camel milk (**b**), soya milk (**c**), and oat milk (**d**) in different levels.

The supervised LDA model, following PCA, was built to discriminate the concentration of adulterated milk and assess the level of adulteration. The correct classification rate was tested with leave-20%-out cross-validation and calculated on the basis of the number of spectra classified correctly compared to all spectra in the full data set. The adulteration levels identified when analysing adulterated each milk groups were; goat milk (5%), camel milk (0.5%), oat milk (0.5%), and soya milk (0.1%) of cow milk content. The LDA model can be used as a straightforward testing model for cow milk adulteration levels in other milk species samples, shown in Fig. 8: (a) goat milk (5–50%), (b) camel milk (0.5–50%), (c) soya milk (0.1–50%), (d) oat milk (0.5–50%). A linear tendency was observed in relation to the adulteration levels in goat-cow, camel-cow, soya-cow, and oat-cow milk adulteration cases.

The camel-cow milk adulteration model (Fig. 8b) has excellent discriminating ability, shown clearly by the level of separation in the LDA model. The detection of 0.5% adulteration level was not feasible, but this is a highly unlikely level of adulteration in commercial fraud as not of economic benefit. The cross-validation of this model gives a correct classification rate of 93.9%. The LDA model for soya milk adulteration with cow milk is shown in Fig. 8c. It can be seen that even soya milk adulterated with 0.1% cow milk can be distinguished from the pure sample. The correct classification rate for this model was 92.2%. A detection limit of 0.5% cow milk adulteration is also clearly revealed in the dairy-free oat milk adulteration model (Fig. 8d), with an 84.3% correct classification rate. The goat milk adulteration model shown in Fig. 8a. Worth noticed that semi-skim goat-cow milk model does not show performance or separation comparable to whole milk model but still can be identified when adulterated with 5% cow milk. That may because most spectrometric features are identical between semi-skim cow milk and goat milk. The correct classification rate for whole goat-cow milk was 82.35% and for semi-skim goat-cow milk was 60.7%. The individual Correct Classification Rate was 100% excluding outliers (standard deviation multiplier-5 σ) and 97.6% when including outliers for five species milk samples. The results indicated the detection of cow milk in milk from different animal and plant sources is feasible using the DESI-MS system.

## Conclusion

Using Desorption Electrospray Ionisation ambient mass for lipids standards characterisation^46^ presents the advantage of analysing many different lipid species directly from samples in one ionisation mode. Moreover, more than 9500 components were found in 5 milk species with DESI-MS. Combined with multivariate analysis, 28 lipids were identified as biomarkers with multivariate analysis. It was observed that the relative abundance of lipid groups in milk from different animal and plant sources appears to be lower than in cow milk; quantification of lipids using a triple-quadrupole instrument would be a logical next step when determining further work to undertake. The discovered milk species-specific biomarkers can be used as presence/absence markers for a given species in trace detection.

In this study, the application of DESI for the rapid lipidomic profiling of milk samples was successfully demonstrated for the first time. This approach has the advantage of providing simultaneous information for milk species identification and can permit the accurate selection of the adulteration levels of screened fraudulent non-cow claimed milk. The profiles show strong specificity in species and in adulteration level identification. Cow milk could be easily distinguished from the dairy-free milk samples (oat milk, soya milk) and milk from different animal sources (camel milk). Goats milk was marginally more challenging but by creating a binary model, it was also easily distinguishable from cow’s milk. The detection limit of these models ranged from 0.1 to ~5% in different milk groups.

In addition to its potential in milk fraud detection, it is an effective quality control method for production factories that may use the same production line for different milk species. The high-level of reproducibility demonstrated by DESI here suggests that if the source can be developed for a simpler, cheaper, and more robust mass spectrometer, such as a single-quadrupole instrument, the technique could be utilised by individuals who have no prior expertise with mass spectrometry. DESI on a more basic instrument is likely to result in a technique that is simple, quick, environmentally responsible, and highly user-friendly.

## Methods

### Samples and reagents

A proof-of-concept study was based on different species of plant milk (oat milk, soya milk) and animal milk (cow milk, goat milk, camel milk). The reproducibility and authenticity studies were performed on 90 cow milk samples. All cow milk samples were purchased from local UK markets and farms, and were directly used without further treatment. For sample measurements, each sample was transferred to a 50 mL headspace vial. Prior to analysis, the samples were stored in the dark at 4 °C in screw cap jars for no longer than 3 days, representative of typical consumer fridge storage conditions.

Cow milk and other species of milk samples were paired randomly and mixed in different proportions [0, 5, 10, 20, 50, 100% (v/v) of cow milk mixed with goat milk, 0, 0.5, 1, 2, 5, 10, 20, 50, 100% (v/v) of cow milk mixed with camel milk; 0, 0.5, 5, 10, 20, 50, 100% (v/v) of cow milk mixed with oat milk, and 0, 0.1, 1, 2, 5, 10, 20, 50, 100% (v/v) of cow milk mixed with soya milk, respectively] to simulate adulterated samples.

Afterward, each sample was diluted with water before testing, milk: water = 1:4 (v: v). The sample solution was directly loaded onto a glass slide sample plate (volume 2 μL, diameter 3 mm) and evaporated to dryness at room temperature (approx. 10 min) for DESI-MS analyses.

LC-MS grade acetonitrile and formic acid (99%) were purchased from Honeywell Riedel-de Haën (Seelze, Germany). Ultra-pure deionised water (18.2 MΩ/cm) was obtained from a Millipore Milli-Q system (Billerica, MA, USA). Micro-24™ slides and Micro-96™ slides were obtained from Prosolia (Indianapolis, IN, USA).

### Instrumentation

Experiments were performed on a Waters G2-XS Q-Tof mass spectrometer (Waters Corporation, Wilmslow, Manchester, UK) fitted with a Prosolia 2D Omni-Spray ion source (Prosolia, Indianapolis, IN, USA) for DESI-MS analysis. Initial setup of the DESI source was performed by the analysis of cow milk using a solvent flow rate of 2 µL/min with N_2_ as a nebulising gas set at 0.7 MPa; the spray solvent was composed of 98% acetonitrile-water (0.2% formic acid included). The spray voltage was set of 4.0 kV and the spray angle of 65°. Prior to analysis, the mass spectrometer was calibrated with 0.5 mM sodium formate solution (90% IPA) infusion flow rate of 5 µL/min, at a mass resolution of 15,000 full width at half maximum (FWHM) at m/z 600. The cone voltage was set at 50 V and the source temperature at 50 °C. Mass spectrometric analysis was performed in positive ion polarity and sensitivity mode over a mass range of 100–2000 m/z with a scan time of 0.5 s/scan. The acquisition time for each sample was 10 s.

### Multivariate data analysis

Mass spectra were collected using MassLynx v4.1 (SCN959) (Waters, Wilmslow, Manchester, UK). The recorded scans for each sample were combined to give an average spectrum and thus one spectrum for each sample was used to build the chemometric models. Raw datasets were analysed with Abstract Model Builder (AMX) v 1.0.1563.0 (Waters Research Centre, Budapest, Hungary). AMX was used to create PCA models, and linear discriminant analysis (LDA) models. All chemometric models were calculated using the mass region of m/z 100–2000, a spectral intensity limit of 1.00E6 counts, and a mass bin width of 0.2 Da. The validation of each model was assessed by the software’s built in “20% out” bootstrapping option. The model was calculated using 80% of the samples and data files left out were classified using the training model.

The multi-variate statistical software package AMX Recognition (Version 0.9.2092.0; Waters Research Centre, Budapest, Hungary) was used to validate and rapidly recognise unknown samples. Four partitions (80%) of the data set were used to build a training model. An outlier threshold (standard deviation) of 5 σ was used for class assignments. A sample will be considered as an “outlier” and excluded from further analysis if the variability exceeds the outlier threshold.

The processed matrix generated within the prototype modelling software was exported to SIMCA 14.1 (Umetrics, Umea, Sweden) allowing the data to be exposed to further chemometric functions such as orthogonal partial least squares-discriminant analysis (OPLS-DA) with the data being mean centred and Pareto scaled. OPLS-DA predictive results visualisation were provided as S-plots and Coefficients vs. VIP. The difference between classes will be shown initially as differences in mass bins, from which the accurate mass of analytes (biomarkers) found within each mass bin can be obtained.

Biomarkers were identified using Lipid Map databases. The instrument was run in MS/MS mode to obtain daughter ions for the identification or confirmation of the chemical structures of biomarkers. Data were acquired in the m/z range of 100–2000 for comparison with the characteristic MS fragmentation patterns from online databases.

### Reporting summary

Further information on research design is available in the Nature Research Reporting Summary linked to this article.

## Supplementary information

nr-reporting-summary-NPJSCIFOOD-00431R1

## Data Availability

The author declared that the datasets generated during the current study are available from the corresponding author on reasonable request.
